# Fructophilic lactic acid bacteria inhabit fructose-rich niches in nature

**DOI:** 10.3402/mehd.v23i0.18563

**Published:** 2012-06-18

**Authors:** Akihito Endo

**Affiliations:** Functional Foods Forum, University of Turku, Turku, Finland

**Keywords:** Fructophilic lactic acid bacteria, fructose-rich niches, Fructobacillus, Lactobacillus kunkeei, adaptation electron acceptor

## Abstract

Fructophilic lactic acid bacteria (FLAB) are a special group of lactic acid bacteria (LAB), which prefer fructose but not glucose as growth substrate. They are found in fructose-rich niches, e.g. flowers, fruits, and fermented foods made from fruits. Quite recently, they were found in the gastrointestinal tracts of animals consuming fructose, which were bumblebees, tropical fruit flies, and *Camponotus* ants. These suggest that all natural sources that are rich in fructose are possible their habitats. *Fructobacillus* spp., formerly classified as *Leuconostoc* spp., are representatives of these microorganisms, and *Lactobacillus kunkeei* has also been classified as FLAB. They share several unique biochemical characteristics, which have not been found in LAB inhabited in other niches. FLAB grow well on fructose but very poor on glucose. These organisms grow well on glucose only when external electron accepters, e.g. pyruvate or oxygen, are available. LAB have been shown to have specific evolution to adapt to their niches and have several niche-specific characteristics. FLAB must have fructophilic evolution during adaptation to fructose-rich niches. FLAB are unique food-related LAB, suggesting a great potential for future food and feed applications.

Lactic acid bacteria (LAB) can be found in various environments including fermented foods and gastrointestinal tracts of human and animals. Fructophilic lactic acid bacteria (FLAB) are a special group of LAB, which like fructose as a growth substrate (1). They are found in fructose-rich niches, e.g. flowers, fruits, and fermented foods made from fruits. *Fructobacillus* spp. are representatives of these microorganisms. They had been originally classified as *Leuconostoc* species (2–4) and later reclassified as *Fructobacillus* species based on their phylogenetic positions and biochemical and morphological characteristics (Fig. 1; 5). *Lactobacillus kunkeei* is only the fructophilic species classified in the genus *Lactobacillus* at present (Fig. 1). This species was originally isolated from wine, and at the time of proposal of the species, its fructophilic characteristics had not been discussed (6). The species was later characterized as FLAB (7). *Lactobacillus florum* shares similar characteristics with FLAB, but it is differentiated from FLAB based on several biochemical characteristics. Thus, this organism is now classified as facultatively FLAB (1, 8).

**Fig. 1:**
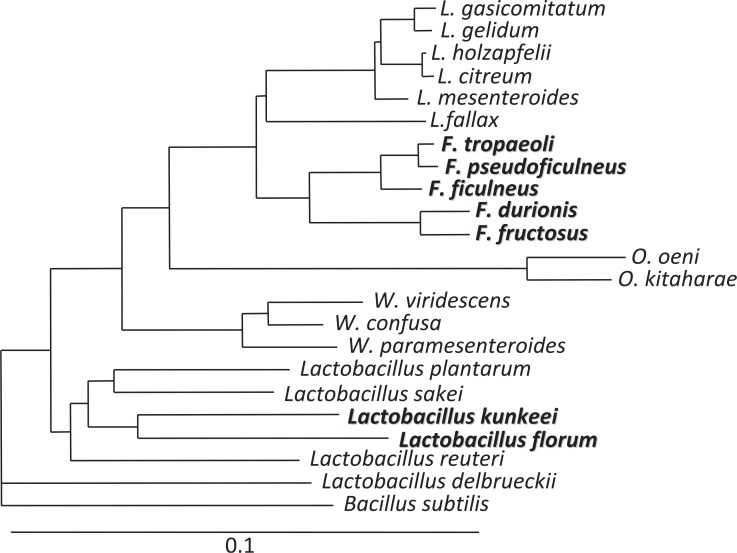
Phylogenetic relationship of FLAB (shown in bold) and phylogenetically related LAB.

## Niche

Fructophilic lactic acid bacteria have been found in various fructose-rich niches until now. *Fructobacillus fructosus*, type species of the genus *Fructobacillus*, was originally isolated from a flower in Japan in 1956 (9), and isolation of the species had not been reported for over 50 years. The species has been isolated from flower, wine, and taberna, a beverage produced in Mexico (1, 10, 11). *Fructobacillus pseudoficulneus* is the most frequently seen *Fructobacillus* species in natural sources, and it has been isolated from figs, bananas, flowers, and taberna (1, 3, 11). *Fructobacillus ficulneus* and *Fructobacillus durionis* have been isolated only from figs and tempoyak (fermented durian condiment), respectively (2, 4). *Fructobacillus tropaeoli* is a recently described species from flowers (12). Recently, a few studies have suggested that cocoa bean fermentation is a rich source of *Fructobacillus* species (13–15). *L. kunkeei* has been isolated from wine, honey, and flowers (1, 7). Interestingly, these FLAB species have also been found in the gastrointestinal tracts of several insects, e.g. bumblebees, tropical fruit flies, and *Camponotus* ants (16–18). Bumblebees and tropical fruit flies are flower-related insects, and their diets are rich in fructose. *Camponotus* ants consume honeydew containing fructose obtained from aphids. These findings suggest that the gastrointestinal tracts of insects consuming large amounts of fructose can be colonized by various FLAB. Such findings are of interest as FLAB prefer aerobic conditions over anaerobic conditions for their growth. The organisms cannot grow under anaerobic conditions if available carbon source is glucose and thus possibly colonize the intestinal tract of vegetarian subjects.

## Biochemical characteristics

**Fig. 2:**
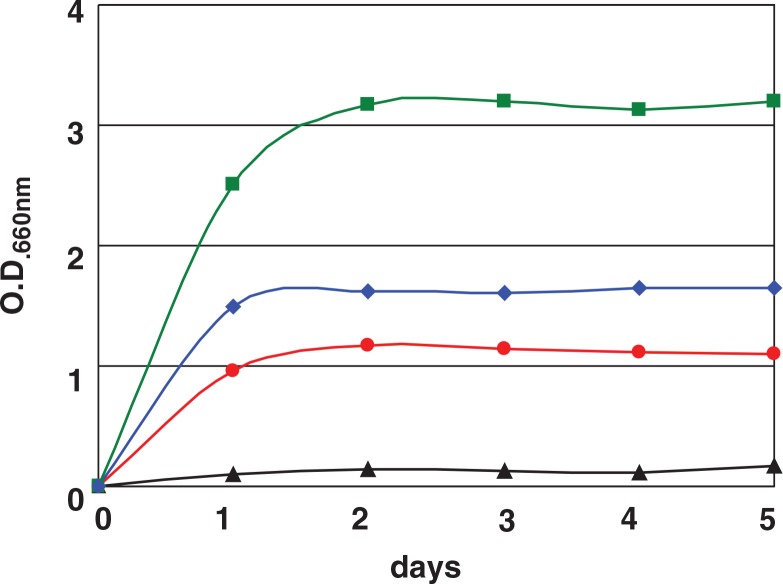
Growth characteristics of *Fructobacillus tropaeoli* F214-1^T^ on fructose (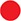), on glucose (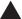), on glucose in the presence of pyruvate (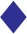), and on glucose under aerobic conditions (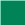).

Fructophilic lactic acid bacteria have very unique biochemical characteristics when compared to other LAB. FLAB grow very well on fructose, but growth is very poor on glucose (Fig. 2), although glucose is the most general substrate for almost all LAB. FLAB produce mannitol from fructose, and they grow well on glucose in the presence of pyruvate or under aerobic conditions (Fig. 2). These results mean that FLAB need electron acceptor for glucose metabolism, and pyruvate and oxygen are used as electron acceptor. These organisms can grow well on agar medium containing glucose as sole carbon source under aerobic conditions but hardly grow under anaerobic conditions (1, 12). These characteristics easily distinguish FLAB from other LAB. Facultatively FLAB species, *L. florum*, grows well on fructose and on glucose in the presence of electron acceptor as same as other FLAB. However, this organism is differentiated from FLAB based on its growth characteristics on glucose without electron acceptor, meaning that *L. florum* can grow on glucose but at delayed growth rate. Based on the carbohydrate fermentation test, FLAB can metabolize only a limited number of carbohydrates (1). They are only two to five. Most of the *Fructobacillus* species can metabolize only fructose, glucose, and mannitol. Metabolism of fructose was done within 1 day, and that of glucose took 2–4 days (1).

All FLAB produce gas from glucose, indicating that they are obligately heterofermentative LAB. However, FLAB are differentiated from other obligately heterofermentative LAB by production of acetate instead of ethanol (1, 5, 7). Obligately heterofermentative LAB normally use phosphoketolase pathway for glucose metabolism. Acetyl phosphate arisen from cleaving of xylulose-5-phosphate by phosphoketolase is converted to ethanol via acetyl-CoA and acetaldehyde in the pathway. AdhE protein coded on *adhE* gene is needed for the conversion (19). However, *Fructobacillus* spp. do not have *adhE* gene (unpublished data). Because of this absence, *Fructobacillus* spp. produce acetic acid instead of ethanol. Conversion of acetyl phosphate to ethanol is an important step to oxidize NADH to NAD, for which NADH is produced in upper stream of the phosphoketolase pathway. Thus, the missing step leads to shortage of NADH for glucose metabolism in the pathway. Therefore, FLAB would need electron acceptors for glucose metabolism. This finding correlates well with their growth characteristics.

## Niche-specific characteristics of LAB

Lactic acid bacteria can be found in various niches, and several studies suggested that LAB have adapted to their niches for their lives (20, 21). Diary LAB, e.g. *Lactobacillus delbrueckii* subsp. *bulgaricus*, *Lactobacillus helveticus*, *Streptococcus thermophilus*, prefer lactose over glucose as growth substrate and are well known to have proteolytic activity (22). Proteolysis is a very important characteristics for dairy LAB as free amino acids are scarce in milk. Gastrointestinal LAB are usually bile tolerant and produce various bacteriocins to compete with other bacteria (23). As FLAB inhabit in fructose-rich niches, they might have lost ability to metabolize glucose during their adaptation to their niches. Genome sequencing of FLAB might clear the evidences of their evolution.

Fructophilic lactic acid bacteria are unique food-related LAB, as described above, suggesting a great potential for future food and feed applications.
